# Flash phase engineering of MoS_2_ nanofilms for enhanced photoelectrochemical performance†

**DOI:** 10.1039/d3ra07759d

**Published:** 2024-02-05

**Authors:** Rong Tan, Yuxin Liu, Yifeng Tu, Felix F. Loeffler

**Affiliations:** a Department of Biomolecular Systems, Max Planck Institute of Colloids and Interface 14476 Potsdam Germany Felix.Loeffler@mpikg.mpg.de; b College of Chemistry, Chemical Engineering and Material Science, Soochow University 215123 Suzhou China tuyf@suda.edu.cn; c Department of Chemistry and Biochemistry, Freie Universität Berlin 14195 Berlin Germany

## Abstract

A heterophase structure combining semiconducting 2H- and metallic 1T-MoS_2_ exhibits significantly enhanced photoelectrochemical performance due to the electrical coupling and synergistic effect between the phases. Therefore, site-selective effective phase engineering is crucial for the fabrication of MoS_2_-based photoelectrochemical devices. Here, we employed a flash phase engineering (FPE) strategy to precisely fabricate a 2H-1T heterophase structure. This technique allows simple, efficient, and precise control over the micropatterning of MoS_2_ nanofilms while enabling site-selective phase transition from the 1T to the 2H phase. The detection of reduced glutathione (GSH) showed an approximately 5-fold increase in sensitivity when using the electrode fabricated by FPE.

## Abstract 2

Flash phase engineering (FPE) enables the fabrication of heterophase MoS_2_ nanofilms that efficiently transition from the 1T to the 2H phase by controlled laser irradiation. This offers large-area fabrication of site-selective heterophase structures.

## Graphical abstract

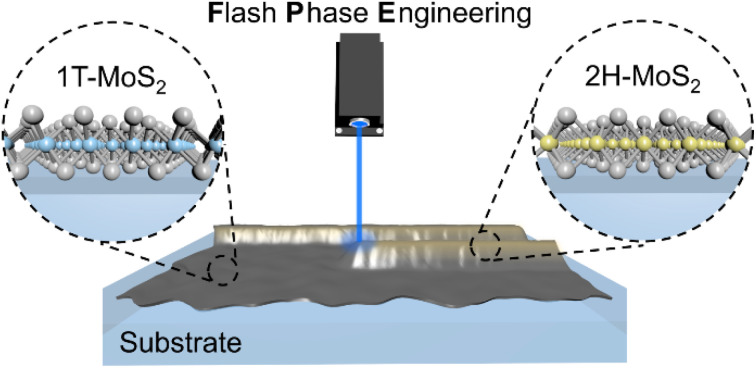

## Introduction

Two-dimensional transition metal dichalcogenides have attracted considerable attention due to their broad potential in electronics, catalysis, biosensors, and energy storage.^1–5^ Among them, molybdenum disulfide (MoS_2_) emerges as the most prominent, featuring a metallic 1T phase and semiconducting 2H phase.^6,7^ While 1T-MoS_2_ is a good electrical conductor, it lacks sufficient optical response capabilities. Conversely, 2H-MoS_2_ exhibits an exceptional optical response but suffers from poor electrical conductivity.^8–11^ These inherent limitations in each phase pose a challenge to their practicality in photoelectrochemical (PEC) devices.^6,10^ To fully exploit the potential of MoS_2_ in PEC applications, it is necessary to couple semiconducting and metallic regions within a single MoS_2_ film by fabricating a 2H-1T heterophase structure.

A direct and effective idea is to drive the partial phase transition in a homophase 1T- or 2H-MoS_2_ film. The *in situ* transition could be realized by electron injection into a thermodynamically stable 2H-MoS_2_ film or annealing treatment of an active 1T-MoS_2_ film.^12–15^ However, both phase transition pathways are challenging in terms of precise control of the extent and location of the phase transition, as they always occur simultaneously throughout the entire film. Therefore, a method to selectively phase transition MoS_2_ by a controllable extent and location would enable tunable electrode properties.

In this work, we propose a practical flash phase engineering (FPE) strategy to fabricate 2H-1T heterophase nanofilms, achieving a controllable 1T to 2H phase transition. 1T-MoS_2_ is a good electrical conductor, but it lacks sufficient optical response capabilities. In contrast, 2H-MoS_2_ exhibits an exceptional optical response for PEC with photogenerated holes. Glutathione (GSH) can act as an electron donor through oxidization in a PEC process, reacting with the holes located on the 2H-MoS_2_. These electrons can be quickly injected into the conduction band by 1T-MoS_2_. The resulting increase in photocurrent enables quantitative detection of GSH.

## Results

**Fig. 1:**
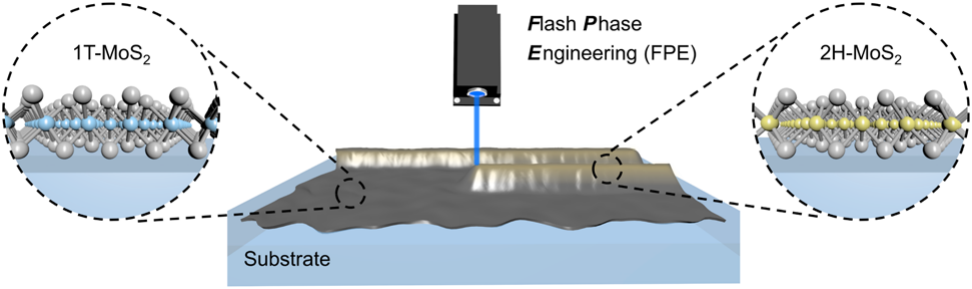
Schematic presentation of 1T to 2H phase transition *via* FPE. A 1T-MoS_2_ nanofilm serves as an absorbing layer, which rapidly converts laser energy into localized thermal energy, triggering its phase transition. The site selective phase transition was achieved by a predefined laser scanning path.

**Fig. 2:**
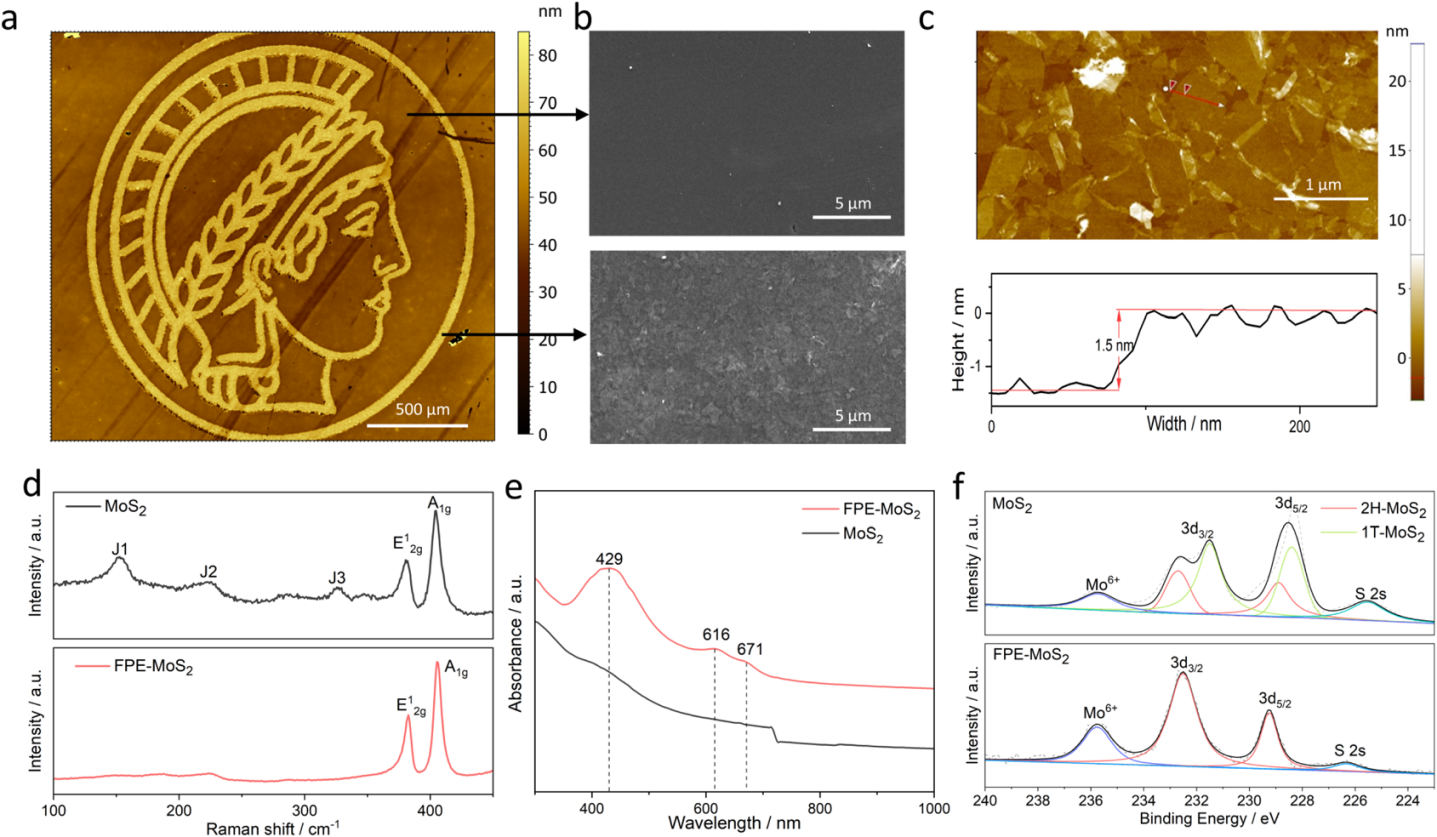
Validation of laser-induced 1T to 2H phase transition. (a) Morphology of the fabricated logo pattern measured by vertical scanning interferometry (VSI). (b) SEM images of MoS_2_ before (top) and after (bottom) FPE. (c) AFM image and thickness profile of exfoliated MoS_2_. (d) Raman spectra, (e) UV-vis absorption spectra, and (f) XPS Mo 3d, Mo^6+^, S 2s spectra of 1T-MoS_2_ and FPE-MoS_2_.

A 1T-MoS_2_ nanofilm was used as the basal substrate, which was obtained *via* wet-chemistry exfoliation and vacuum filtration with a 25 nm pore size membrane.^14^ Different nanofilm thicknesses were controlled by adjusting the volume of 1T-MoS_2_ during the fabrication process. Then, the desired micro-/nanopattern coupling of two phases was achieved by FPE (Fig. 1). A 488 nm laser beam with a focus diameter of 15 μm (1/*e*^2^) was employed, and its power and scan speed were flexibly tuned for precise control.^15,16^ For example, a logo pattern was achieved by a predefined laser scan path (Fig. 2a). A distinct contrast could be observed in the scanning electron microscope (SEM) image before and after the laser treatment (Fig. 2b). The atomic force microscopy (AFM) image and thickness profile show the ultrathin (nanometer) thickness of the fabricated heterophase MoS_2_ nanofilm (Fig. 2c).

Raman and UV-vis spectroscopy were used to verify the laser-induced phase transition in MoS_2_. The Raman spectra show characteristic peaks originating from the in-plane (E^1^_2g_) and out-of-plane (A_1g_) Mo-S phonon modes both before and after laser treatment (Fig. 2d). However, in the flash phase engineered MoS_2_ (FPE-MoS_2_), the specific peaks corresponding to 1T-MoS_2_ are absent, designated as J_1_ (153.2 cm^−1^), J_2_ (225.1 cm^−1^), and J_3_ (327.9 cm^−1^) modes, indicating a phase transition from 1T- to 2H-MoS_2_.^14,17,18^ The UV-vis absorption spectrum of FPE-MoS_2_ clearly shows 2H-MoS_2_ excitonic features (Fig. 2e). A and B excitonic peaks were observed at approximately 616 nm and 671 nm, respectively, while C and D excitonic peaks appeared at around 429 nm.^14^

To further validate the phase transition, X-ray photoelectron spectroscopy (XPS) was used (Fig. 2f). Two characteristic peaks were observed in both MoS_2_ and FPE-MoS_2_ at approximately 232.5 eV and 228.9 eV, corresponding to Mo 3d_3/2_ and Mo 3d_5/2_, respectively. Additional peaks at 231.4 eV and 228.2 eV could be assigned to 1T-MoS_2_, while peaks at 232.5 eV and 228.9 eV suggest the presence of the 2H phase.^6,19^ In addition, all Mo 3d and S 2p peaks of the FPE-MoS_2_ sample shifted to a slightly higher binding energy (Fig. S1†). The appearance of all of the above features indicates the successful laser-induced 1T to 2H phase transition.

**Fig. 3:**
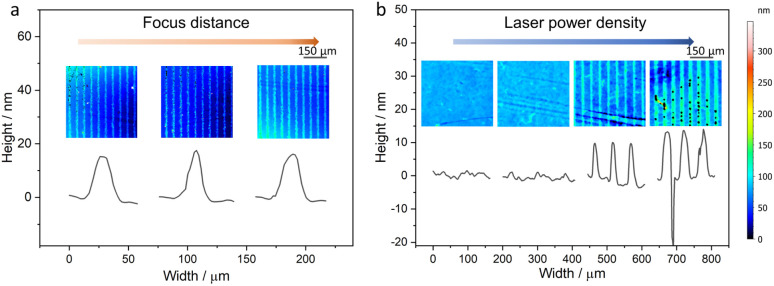
Optimization of laser parameters. Microline pattern fabricated using different (a) focus distances (4.08, 4.18, 4.28 mm from left to right). Here, 4.18 mm was defined as 0 and the other two values are offset by 100 μm (b) different laser power densities of 0.063, 0.227, 0.456, and 0.631 mW μm^−2^ were screened from left to right.

In the FPE process, the incident laser beam irradiates the 1T-MoS_2_ nanofilm, which serves as an absorbing layer, resulting in a rapid conversion of laser energy into localized thermal energy. Consistent with our previous investigations, the estimated temperature at the center of the laser irradiation spot on each absorber exceeds 343 °C (Fig. S2†),^20^ which triggers the phase transition process.^21^ The precise location of the laser focus was optimized to hit the nanofilm interface (Fig. 3a).

It should be noted that the thickness of the 1T-MoS_2_ substrate nanofilm also influences the effect of phase transition, as evidenced by the patterns observed in a simple microline design (Fig. 3b and S3†). The sharpness/clarity of the lines increases with thicker layers and higher laser power densities, indicating that more MoS_2_ undergoes phase transition, until some defects start to emerge at ≥0.631 mW μm^−2^. This phenomenon is more pronounced in the PEC performance, which is strongly influenced by the 2H-1T heterophase structure. FPE shows minimal PEC enhancement on the 20 nm 1T-MoS_2_ nanofilm, while it becomes more pronounced on the thicker 1T-MoS_2_ nanofilms. In this context, the 1T-MoS_2_ nanofilm serves as an effective charge carrier acceptor and transporter, suppressing the recombination of photogenerated charge carriers and facilitating their rapid transport. Consequently, this leads to enhanced PEC performance due to the larger number of charge carriers effectively contributing to the photoelectrochemical process.

**Fig. 4:**
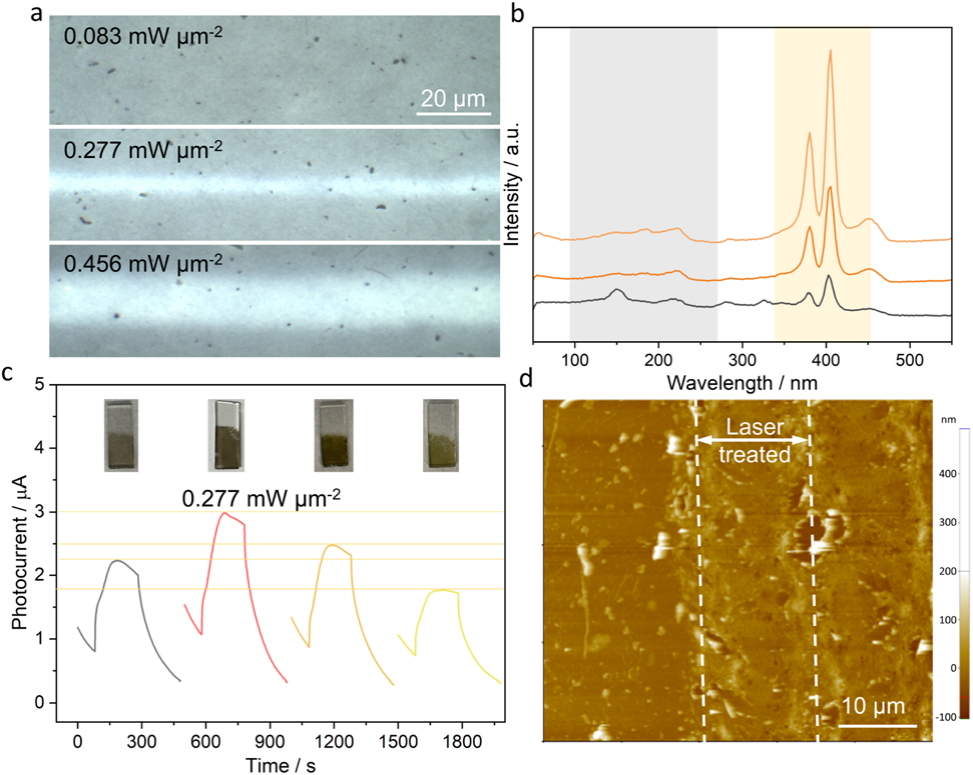
FPE-MoS_2_ device with enhanced PEC performance. (a) Optical images of FPE-MoS_2_ under different laser power densities for phase transition. The corresponding (b) Raman spectra and (c) photocurrent response with optical images of FPE-MoS_2_ electrodes inserted under 0.5 V *vs.* Ag/AgCl under white light irradiation. (d) Atomic force microscopy image of optimal FPE-MoS_2_, using 0.277 mW μm^−2^ laser power density, 10 mm s^−1^ laser scan speed, and 0.05 mm line spacing.

In addition, due to the more obvious difference in PEC response between different laser densities, the 35 nm thin 1T-MoS_2_ nanofilm was used as a model platform to study the effect of the laser-induced flash phase transition and the underlying mechanisms driving the enhancement of PEC performance. As the laser power density increases, a greater amount of 1T-MoS_2_ undergoes phase transition to the 2H-MoS_2_ (Fig. 4a), as evidenced by the Raman spectra showing weaker peaks in the 1T phase and stronger peaks in the 2H phase (Fig. 4b). The photocurrent response shows an initial increase and then decreases with increasing laser power density, reaching a maximum at 0.277 mW μm^−2^ (Fig. 4c). This behavior can be mainly attributed to the degree of phase transition to 2H-MoS_2_, which has a high optical absorption but a limited charge transfer capability. Less 2H-MoS_2_ (potentially a mono- or few layer transition) was achieved under very low irradiation (0.083 mW μm^−2^), which suffers from low light absorption since it is too thin (as shown in Fig. 4a). In contrast, too much 2H-MoS_2_ in the film hinders electron transport, leading to diminished PEC performance. Thus, the introduction of moderate amounts of 2H-MoS_2_ within the 2H-1T heterophase structure provides a favorable balance between optical absorption and efficient electronic transport. In addition, other laser parameters were also studied, such as laser scan speed and line spacing (Fig. S4†). Specifically, the optimized PEC performance was achieved under the laser power density of 0.277 mW μm^−2^, 10 mm s^−1^ laser scan speed, and 0.05 mm line spacing. Additionally, the FPE process results in thermal and morphological folding (Fig. 4d), increasing the specific surface area of the electrode for enhanced optical absorption.

**Fig. 5:**
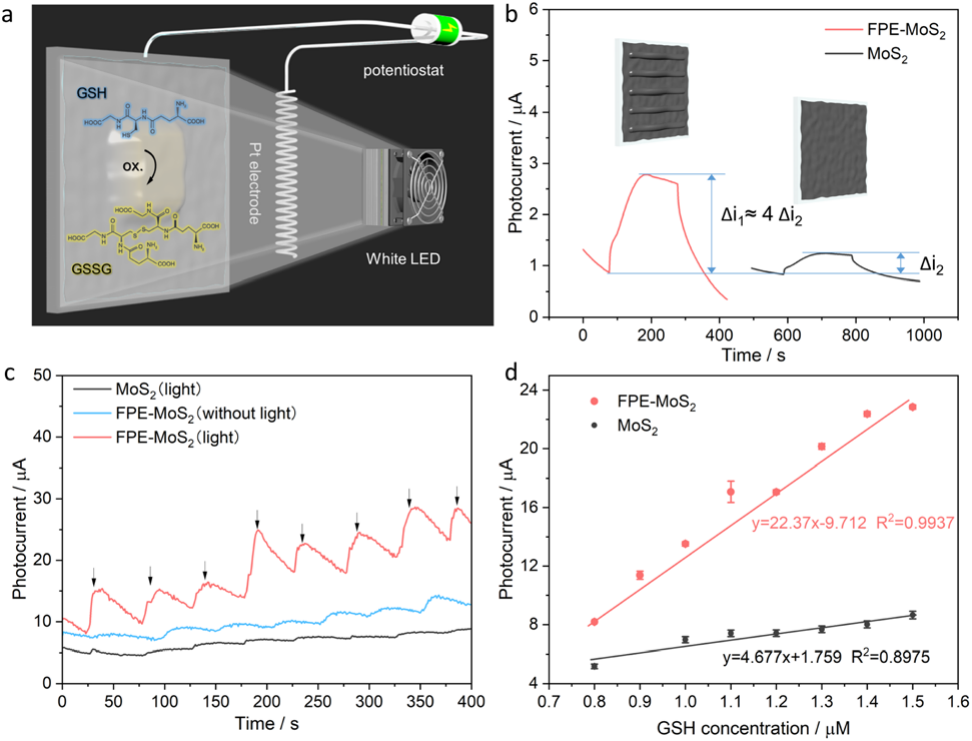
Biosensing application of FPE-MoS_2_ PEC device. (a) Scheme of the PEC device with MoS_2_ on FTO as the photoanode and Pt as the photocathode. (b) Photocurrent response of FPE-MoS_2_ (Δ*i*_1_) and MoS_2_ (Δ*i*_2_) electrodes under 0.5 V *vs.* Ag/AgCl under white light irradiation. (c) Amperometric responses of relative electrodes under the same PEC conditions for successive additions of GSH. (d) Corresponding calibration curves for the MoS_2_ and FPE-MoS_2_ electrodes with white light illumination.

Then, this optimized FPE-MoS_2_ structure was used as a photoanode in a PEC device, as shown in Fig. 5a. The results show that the photocurrent of the optimized FPE-MoS_2_ electrode exhibits a substantially higher response (Δ*i*_1_) compared to the non-engineered counterpart (Δ*i*_2_) during the on–off cycles of white light irradiation (Fig. 5b). Finally, the optimized PEC device was used for biosensing applications. In this study, reduced GSH was used as a model analyte due to its relevance in various diseases, including aging and cancer.^22,23^ To evaluate the biosensing activity of the 2H-1T heterophase structure, chronoamperometry (*i*–*t*) measurements (Fig. 5c) were conducted. The results show a substantial increase in the photocurrent response with increasing GSH concentration, specifically at 100 nM intervals. Upon the addition of GSH to the system, the reducing agent serves as the electron donor, which quickly scavenges the holes, suppressing the recombination of photogenerated charge carriers. These electrons were injected into the conduction band of the MoS_2_ and subsequently transferred to the FTO electrode, leading to a relatively large photocurrent response. During this process, GSH is oxidized to glutathione disulfide. The 1T-MoS_2_ electrode with light was compared with the FPE-MoS_2_ electrode with and without light, which highlights the significant enhancement in biosensing of the 2H-1T hetero-phase electrode under light irradiation. Further examination revealed that the photocurrent exhibited a linear relationship with physiological GSH concentrations (Fig. 5d). This linear relationship demonstrates high sensitivity (*S* = Δ*i*/Δ*c*), which is approximately five times higher than that observed in MoS_2_ biosensing systems without phase engineering. In addition, the stability was studied with an electrode which was kept at 4 °C, periodically measuring its performance (Fig. S5a†). After 7 days, the photocurrent response of the FPE-MoS_2_ electrode decreased by less than 13%, indicating good storage stability. Moreover, the reproducibility of the optimized PEC device was verified by measuring the photocurrent response of three electrodes in parallel under successive addition of GSH, showing an RSD of 5.36% (Fig. S5b†). This, indicates a high reproducibility of the FPE-MoS_2_ electrode.

## Conclusions

This study demonstrates a facile and efficient method to achieve a controllable 1T to 2H phase transition in a MoS_2_ nanofilm through FPE. The fabrication of a 2H-1T MoS_2_ heterophase structure has led to a significant improvement in PEC performance, which is contributed to the synergistic coupling of metallic 1T-MoS_2_ and semiconducting 2H-MoS_2_ phases. The inclusion of 2H-MoS_2_ as a light absorber and the utilization of 1T-MoS_2_ as an excellent electron acceptor and transporter collectively enhance the photoelectrochemical activity of the material. This work introduces a practical strategy to achieve the phase transition and offers promising potential for large-area fabrication of site-selective heterophase structures. Moreover, the findings open up opportunities for a wide application range, including biosensing. The ability to control the phase transition in MoS_2_ through this approach may pave the way for advanced biosensing platforms with improved sensitivity and performance, facilitating early disease diagnostics and other (bio)analytical applications.

## Author contributions

The manuscript was written through contributions of all authors. All authors have given approval to the final version of the manuscript.

## Conflicts of interest

The authors declare no competing financial interest.

## Supplementary Material

RA-014-D3RA07759D-s001
