# Supplementary material for: Flash phase engineering of MoS2 nanofilms for enhanced photoelectrochemical performance
Source: RSC Adv. 2024 Feb 5;14(7):4730–3. doi: 10.1039/d3ra07759d (PMC10839749; doi:10.1039/d3ra07759d)
Supplement: RA-014-D3RA07759D-s001 [file RA-014-D3RA07759D-s001.pdf]

Electronic Supplementary Material (ESI) for RSC Advances.
This journal is © The Royal Society of Chemistry 2024

Supporting information

Flash Phase Engineering of MoS_2 Nanofilms for Enhanced Photoelectrochemical Performance

Rong Tan[1, 2], Yuxin Liu[1, 3], Yifeng Tu[2, *], Felix Löffler[1, *]

[1] Department of Biomolecular Systems, Max Planck Institute of Colloids and Interface, 14476 Potsdam, Germany

[2] College of Chemistry, Chemical Engineering and Material Science, Soochow University, 215123 Suzhou, China

[3] Department of Chemistry and Biochemistry, Freie Universität Berlin, 14195 Berlin, Germany

Experimental section

Preparation of 1T-MoS_2. The 1T-MoS_2 was obtained by exfoliation of bulk MoS_2 *via* Li intercalation according to the literature [1]. In brief, 300 mg of MoS_2 powder was immersed in 5 mL hexane containing 1.6 M *n*-butyllithium for 48 h. Then, excess *n*-butyllithium was removed by washing with anhydrous ethanol for three times. The resulting solids were subsequently immersed in 45 mL water. To accelerate the delamination, the solution was shaken manually for 15 min and sonicated for an additional 15 min. The resulting solution was centrifugated at 6000 rpm for 30 min to remove the unexfoliated MoS_2 and multi-layers of MoS_2 (repeated 3 times). Another centrifugation step at 11000 rpm for 30 min was performed to remove nanoparticles resulting from side reactions and small-sized MoS_2. Finally, the resulting solution was degassed with Ar for 30 min and stored in the refrigerator at 4 °C. The concentration was quantified as 0.5 mg mL^{-1} after vacuum drying.

Fabrication of 1T-MoS_2 substrate nanofilm. 1T-MoS_2 substrate nanofilm was obtained *via* wet-chemistry exfoliation and vacuum filtration with a 25 nm pore sized membrane (Merck, MCE-Millipore). By adjusting the volume during the fabrication process, nanofilms of different thicknesses could be achieved.

Laser direct writing setup. The laser direct writing setup is a 200 mW TOPTICA iBeam smart 488-S laser with a wavelength of 488 nm (TOPTICA Photonics AG, Germany) [2]. The laser setup can be flexibly tuned in scanning speed and laser intensity, with a $1/e^2$ focus spot of 15 μm.

Fabrication of 1T-2H MoS_2 heterophase. 1T-2H MoS_2 heterophase was obtained by FPE. The laser set up was employed for precise control 1T and 2H phases by flexibly tuning its power density, scan speed and other parameters.

Photoelectrochemical (PEC) measurements and GSH detection. The chronoamperometry curves (i-t) were measured with an electrochemical potentiostat (Gamry, USA) with a three-electrode system in 0.5 M Na_2SO_4 solution with Pt as counter electrode and Ag/AgCl as reference electrode, under a fiber-coupled white LED lamp (455 nm, 300 mW cm^{-2}). The working electrode was achieved by FPE on 1T-MoS_2 substrate nanofilm under different laser parameters. The response of MoS_2 and FPE-MoS_2 electrodes to GSH was evaluated by successively adding 100 nM GSH solution every 50 s. Measurements were performed at 0.5 V vs. Ag/AgCl electrode under white light irradiation.

Instrument. Ultraviolet-Visible (UV-Vis) absorption spectra were measured on a SHIMADZU UV- 3600i Plus UV-vis-NIR spectrophotometer. Atomic Force Microscopy (AFM) measurement was conducted on a Park NX10 AFM with tapping mode. Raman spectroscopy was conducted on a WITec alpha 300 R-Raman Imaging Microscope with the excitation at 532 nm, the diameter of focus spot is about 8 μm, which is centered at the printed lines. X-ray photoelectron spectroscopy (XPS) was measured on an ESCALAB 250Xi spectrometer.

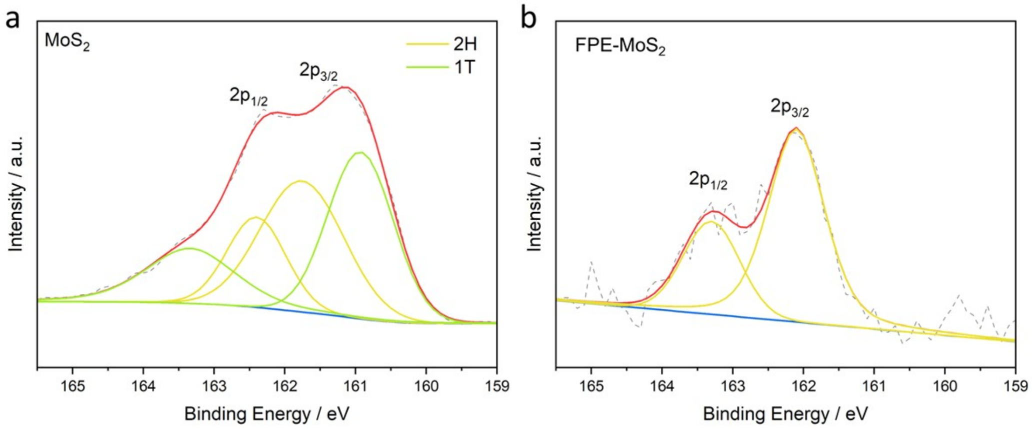

Figure S1. XPS S 2p spectra of MoS_2 and FPE-MoS_2.

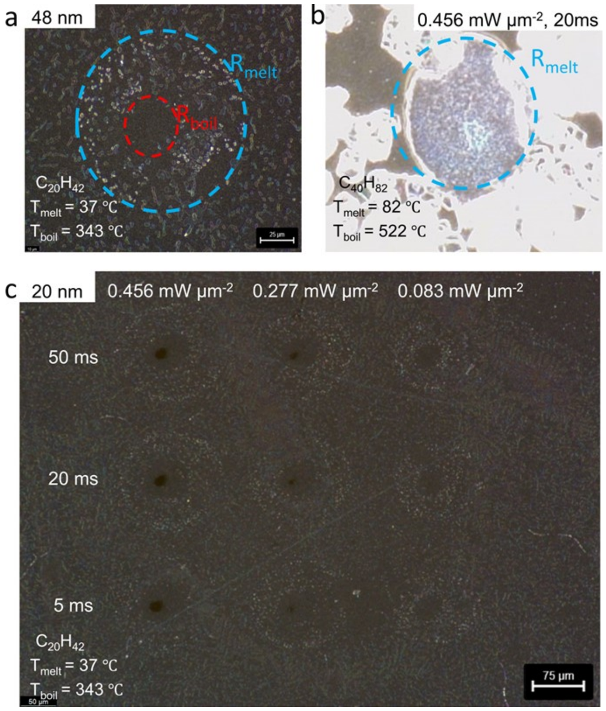

Figure S2. Investigation of laser-induced MoS_2 surface temperature. Light microscopy image of a 0.456 mW µm^{-2} laser irradiation (20ms) on a 48 nm thick MoS_2 absorber with (a) a $C_{20}H_{42}$ alkane thin film and (b) a $C_{40}H_{82}$ alkane thin film. (c) Different laser irradiation parameters on 20 nm thick MoS_2 absorber, coated with a $C_{20}H_{42}$ alkane thin film.

Here, the shorter chain alkane forms a melting ring (R_{melt}) and a rupture hole (R_{boil}) on 48 nm thick MoS_2 absorber after the laser irradiation, while the longer chain alkane only shows a melting ring (R_{melt}). According to the boiling temperature of $C_{20}H_{42}$ (373 °C) and $C_{40}H_{82}$ (522 °C), the temperature on a 48 nm thick MoS_2 absorber could be estimated between 373 and 522 °C. Overlapping of the corresponding melting and boiling rings both appear on the 20 nm thick MoS_2 absorber, so that the temperature on all MoS_2 absorbers could be confirmed to reach >343 °C in the spot center.

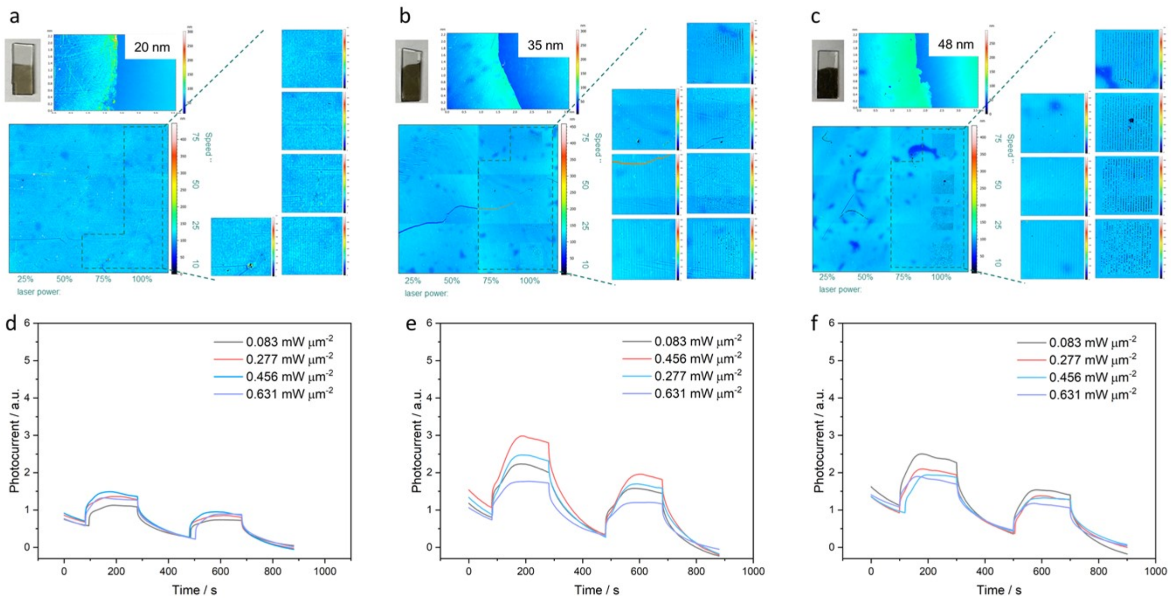

Figure S3. (a, b, c) Optical images and vertical scanning interferometry (VSI) measurements of MoS_2 films of different thicknesses and line patterns fabricated with different laser parameters. (d, e, f) Corresponding photocurrent responses at 0.5 V *vs*. Ag/AgCl under white light irradiation.

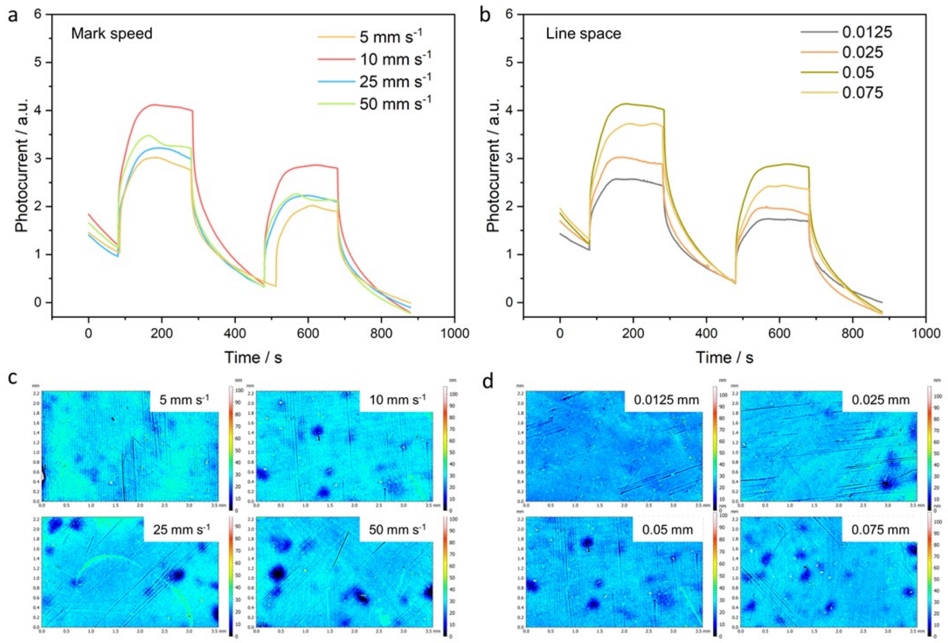

Figure S4. Photocurrent responses on 35 nm thin MoS_2 film, processed with 0.277 mW μm^{-1} laser irradiation at (a) different laser mark speeds and (b) different laser pattern line spacing, at 0.5 V *vs.* Ag/AgCl under white light irradiation. (c, d) Corresponding vertical scanning interferometry (VSI) images.

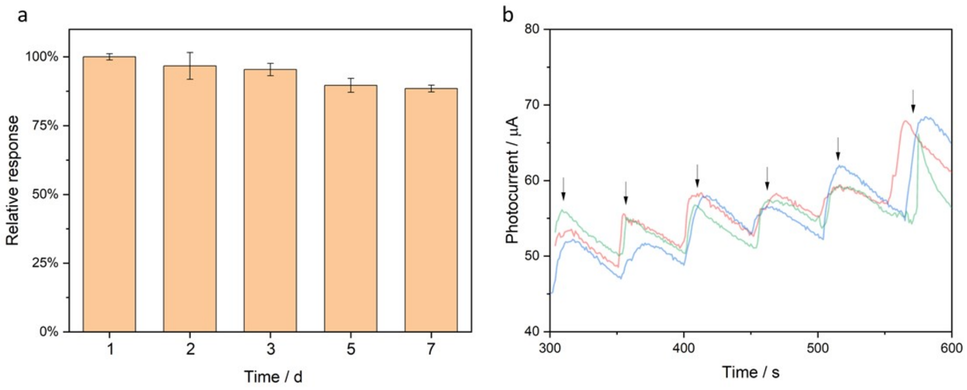

Figure S5. (a) Stability measurements of the resultant FPE-MoS_2 electrode over 7 days. (b) Reproducibility measurement of three different electrodes.

References

[1] Eda, G.; Yamaguchi, H.; Voiry, D.; Fujita, T.; Chen, M. W.; Chhowalla, M. *Nano Lett.*, **2011**, 11, 5111-5116.

[2] Zhang, J.; Zou, Y.; Eickelmann, S.; Njel, C.; Heil, T.; Ronneberger, S.; Strauss, V.; Seeberger, P. Savateev, A.; Loeffler, F. *Nature Communication*, **2021**, 12, 3224.
